# Retraction: The biofilm formation and antibiotic resistance of bacterial profile from endotracheal tube of patients admitted to intensive care unit in southwest of Iran

**DOI:** 10.1371/journal.pone.0320615

**Published:** 2025-03-12

**Authors:** The PLOS One Editors

The *PLOS One* Editors retract this article [1] due to concerns about compliance with the PLOS Human Subjects Research policy.

The corresponding author (AAZD) reported that the published ethics approval number is incorrect, and they requested a revision of this number to IR.AJUMS.REC.1399.848.

The editors do not consider the ethics approval documentation provided in post-publication discussions to be sufficient to confirm compliance with the PLOS Human Subjects Research policy. The editors regret that this issue was not identified prior to publication.

AAZD did not agree with the retraction. ZD, AAH, AFS, NAK, SSF, MMotahar, FJM, FA, HM, PB, RH, and MMoradi either did not respond directly or could not be reached.
